# Timely development of vaccines against SARS-CoV-2

**DOI:** 10.1080/22221751.2020.1737580

**Published:** 2020-03-08

**Authors:** Shan Lu

**Affiliations:** Laboratory of Nucleic Acid Vaccines, Department of Medicine, University of Massachusetts Medical School, Worcester, MA, USA

The rapidly emerging SARS-2-CoV (SARS-2) has been spreading through China and entering many parts of the world with easy human-to-human transmission and thousands of deaths [1–10]. Development of a vaccine, and a vaccine which can be quickly deployed on a global scale, is no longer merely a discussion or part of a debate whether such a vaccine is ultimately needed.

## Why is a vaccine needed?

While the first wave of this outbreak appears to be under control in many parts of China, there are a number of remaining concerns:

1. When will new cases of infection stop emerging from the “ground zero” Wuhan City? Since the real origin of infection is still not fully confirmed, a complete control of infection in Wuhan is essential for a sustained control in the whole country.
2. Will there be any near-term resurging of cluster cases in other parts of China, including regions in Hubei beyond Wuhan, capital of the province? The random cases can be quickly put under control given the highly alert local monitoring systems and well-practiced healthcare teams’ effective work over the last several weeks. But the cluster cases may pose a new threat for their potential to generate another regional outbreak.
3. Will the world, especially those countries less prepared or with less healthcare resources, be able to handle the sudden appearance of cases at their doorstep? The assumption of persistent transmission of SARS-2 at a global scale may no longer be so far-fetched. Even if the virus does not bring a high mortality, the scenario of Community Acquired Coronavirus Infection (CACI) caused by a SARS type virus as part of our daily life will likely disrupt the world social and economic order.

The same calls for vaccines against SARS and Zika faded after the peak of those mysterious outbreaks, partially due to no public demand or limited commercial return to the vaccine investment. However, a timely development of vaccines against SARS-2 is needed this time, not only for controlling the infection but also for stabilizing the global mood and bringing the economy back on track.

## The current efforts to quickly develop a SARS-2 vaccine

The good news now is that many entities have taken actions. CEPI (Coalition for Epidemic Preparedness Innovations) [11] announced on January 23, 2020, the funding to three platform vaccine technologies, DNA, mRNA, and “molecular clamp”, to develop vaccines against SARS-2. CEPI's mission is to “accelerate the development of vaccines against emerging infectious diseases and enable equitable access to these vaccines for people during outbreaks.” The current outbreak will be the first major test to CEPI since its establishment in 2017.

There is a growing list of global public and private institutions joining the efforts to develop vaccines against SARS-2. The US NIAID Vaccine Research Centre (VRC) is drawing on broad research experience with coronaviruses, combined with a wide network of collaborators from academia, other government agencies, and industry, on the development of various SARS-2 vaccine candidates. Biotech and traditional vaccine companies in many countries announced their plans to quickly develop vaccines using their respective technologies.

From very early in the onset of this outbreak, China demonstrated confidence in developing a vaccine against this viral infection. More recently, the official announcement by the Chinese Health Commission indicated that at least five vaccine technologies will be explored: inactivated vaccine, subunit protein vaccine, nucleic acid vaccine, adenoviral vector vaccine, and recombinant influenza viral vector vaccine. With the growing size of the domestic vaccine industry in recent years, China vaccine developers are expected to announce multiple leading candidate vaccines in the near future.

## What are the challenges

The vaccine industry based on the empirical technology has made major contributions to human health over the last 100 years. However, vaccine science is still young in light of the modern immunology and molecular microbiology which have contributed to the requirement of a longer time to develop a new vaccine. Enhanced safety concerns, increasingly complicated manufacturing processes, and related assay requirements are adding to the time and cost for new vaccine development. A new set of rules and standards will need to be adopted to balance the competing scientific, technical, regulatory, and public health considerations, if a quick response SARS-2 vaccine needs to be developed for near future clinical use.

In recent years, immune correlates of protection are increasingly asked for a candidate vaccine [12]. Structure-guided antigen design is quite common. At the same time, vaccine development is still far from being a perfect science. The discontinuation of HVTN702 reported within the last few weeks reminded us again of the big gap between science and the development of an HIV vaccine after almost four decades’ effort. Will protective antibody responses be the targeted immune responses for various vaccine programs against SARS-2? Given the potential challenge in quickly organizing efficacy studies, the animal models against challenge will be extremely valuable for selecting the candidates into humans.

Traditional vaccine technologies need improvement and a wide variety of new technologies have emerged in the last two decades [13]. Given the unique requirements of a vaccine against the rapidly spreading emerging viral infection, vaccine technologies with previous human study experience will have the advantage, especially for the consideration of safety. Furthermore, whether the developer can quickly move its vaccine technology into a scale-up GMP production for potentially 10-million doses is another challenge. Anyone with an existing facility and the experience of such production will be in a much more favourable position.

The challenge to the regulatory agencies for a timely SARS-2 vaccine is similar to that of the vaccine developers. Safety evaluation of a candidate vaccine against SARS-2 will receive high-level attention. The immunopathogenesis plays a major role in SARS-2 infection and thus it is important to ensure that vaccines against this virus should not elicit the same type of detrimental immune responses. This will affect the type of vaccines to be selected and immunogens to be designed. Will a qualified manufacturing process be sufficient for the advancement of a candidate vaccine or it has to be validated? Can the manufacturing and regulatory experience achieved in other countries be applied to the review of a vaccine application in the current country? Can the cell banks or other intermediate products be accepted across country borders? Will the political or commercial considerations become the barriers to the global effort in addressing the urgent need of a SARS-2 vaccine?

Finally, the planning should start now on how to let the world have equal access to a successful SARS-2 vaccine if the need is global. The following issues need to be addressed: vaccine ownership, the funding for production at an unprecedented scale, the pricing and supply chain, and the coordinated administration of such a vaccine to achieve the best outcome of full control of the endemic.

## A vaccine beyond the current outbreak

Even if the global spread of SARS-2 is finally under control before a successful vaccine is fully developed, the vaccine community and world public health leaders need to decide whether a SARS-2 vaccine still should be licensed to serve as a template for vaccines in the future to prevent the outbreaks of other SARS-like viruses. Within 18 years, the world has witnessed three major emerging pathogenic beta coronaviruses (SARS, MERS, and SARS-2) entering large human populations. It may not be too ambitious to suggest a vaccine that can provide broad coverage against more than one of these pathogenic viruses to prevent for future outbreaks.

It is generally agreed that developing a new vaccine needs many years’ efforts. However, the development of vaccines in recent years against EV71, a highly pathogenic virus causing severe Hand, Foot and Mouth Disease (HFMD) in children, has provided a good example that it is possible to develop a vaccine against a modern emerging infection [14,15]. Hopefully, it can be even faster this time and possibly using newer vaccine technologies.
